# Data Uploading Strategy for Underwater Wireless Sensor Networks

**DOI:** 10.3390/s19235265

**Published:** 2019-11-29

**Authors:** Xiangdang Huang, Shijie Sun, Qiuling Yang

**Affiliations:** 1 School of Computer Science and Cyberspace Security, Hainan University, Haikou 570228, China; xiangdanghuang@126.com; 2 State Key Laboratory of Marine Resource Utilization in South China Sea, Hainan University, Haikou 570228, China; 3 School of Information and Communication Engineering, Hainan University, Haikou 570228, China; sun@hainanu.edu.cn

**Keywords:** underwater wireless sensor networks, data uploading decision-making strategy, energy consumption

## Abstract

Underwater wireless sensor networks (UWSNs) have become a popular research topic due to the challenges of underwater communication. The existing mechanisms for collecting data from UWSNs focus on reducing the data redundancy and communication energy consumption, while ignoring the problem of energy-saving transmission after compression. In order to improve the efficiency of data collection, we propose a data uploading decision-making strategy based on the high similarity of the collected data and the energy consumption of the high similarity data compression. This decision-making strategy efficiently optimizes the energy consumption of the networks. By analyzing the data similarity, the quality of network communication, and uploading energy consumption, the decision-making strategy provides an energy-efficient data upload strategy for underwater nodes, which reduces the energy consumption in various network settings. The simulation results show that compared with several existing data compression and uploading methods, the proposed data upload methods has better energy saving effect in different network scenarios.

## 1. Introduction

**Figure 1:**
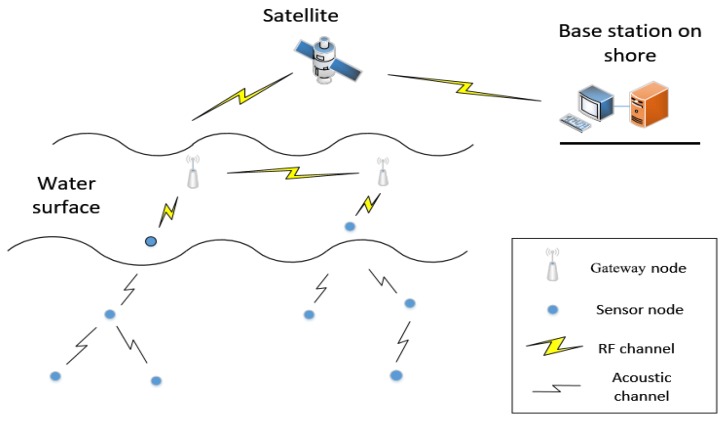
Underwater wireless sensor networks (UWSNs).

Underwater sensor networks (UWSNs) [1] deploy sensor nodes to communicate and simplify computing functions in the monitored area, as shown in Figure 1. The nodes communicate through underwater acoustics and form an Ad Hoc Network. The data are collected by carrying sensors, such as conductivity sensors, temperature sensors, depth sensors, and so on. It enters maritime network through intelligent data gateways or other offshore based stations. UWSNs have made many achievements in underwater environment exploration, such as marine data collection, water quality monitoring, and marine ranch prediction. 

Underwater long-range transmission can only be achieved by using acoustic waves. However, the energy consumption of acoustic communication is very high. Due to cost, underwater nodes are usually powered by batteries. Large-scale and high-density deployments or special underwater application environments increase the difficulty of replacing batteries. Therefore, reducing the energy efficiency has become the primary design goal of UWSNs design.

For large-scale underwater monitoring applications, nodes in UWSNs forward the collected data to the sink node through multi-hop routing. In the actual application environment, many raw data have spatial-temporal redundancy [2]. Therefore, the redundant network processing can reduce the energy consumption of network communication by minimizing the amount of data transmitted, and on the premise of preserving the information required by the application. In the energy-optimized protocol design of UWSNs, data compression is an important aspect of data processing within the network.

**Figure 2:**
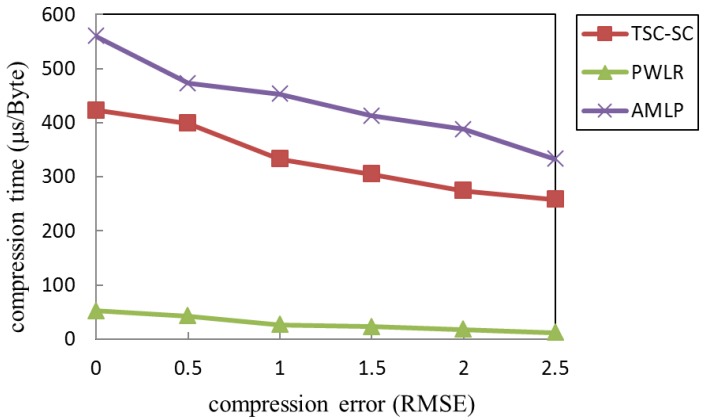
Relationship between compression error and compression time.

Compression algorithms applied to wireless sensor networks have put forward the basic requirements in terms of complexity and compression effect. In the article [3], compression precision and compression ratio are used as the performance indicators to evaluate the compression effect of a data compression algorithm based on data correlation applied in the wireless sensor network (WSN), including the two-step data compression algorithm based on sequence correlation (TSC-SC) [4], adaptive multiple-modality data compression algorithm based on lifting wavelet and adaptive polynomial fitting (AMLP) [5], and data compression algorithm based on piece-wise linear regression (PWLR) [6]. The computational complexity of these algorithms be satisfied by the UWSNs node hardware configuration can satisfy the computational complexity of these algorithms. These algorithms are all based on a time-domain compression algorithm, and the compressed data is sent after the accumulation of local compressed data. The compression accuracy is adjustable. These features ensure algorithm is suitable for the most sensor network applications, including UWSNs. As shown in the simulation results [3], under the same compression accuracy requirements, the AMLP algorithm produces a lower compression ratio, followed by TSC-SC, while the PWLR algorithm produces the highest compression ratio. To further analyze the time complexity of each algorithm, we used ATMEL AVR Studio 4 [7] to compile the algorithms to get the time cost of each algorithm, as shown in Figure 2.

Considering the data collected by a single node, the PWLR has significantly less compression time than other algorithms due to fewer execution steps and computations. However, its compression ratio is significantly lower than the AWLP, which is based on the lifting wavelet and the polynomial fitting. Shuang et al. [4] analyzed the compression energy consumption of the TSC-SC algorithm based on the polynomial fitting. When the fitting number is three, the algorithm shows good performance and the communication energy consumption is further reduced. However, its energy-saving effect is unsatisfactory, which means the computational energy consumption of the algorithm cannot be ignored. The communication energy consumption saved by data compression may not compensate for the additional computational energy consumption introduced, resulting in an increase in total energy consumption. 

As shown in Table 1, the underwater nodes are not only limited in energy, but also the computing power. The time it takes for a node to send single-byte data is about 0.8–8 ms [1]; When calculating a more complex implementation of the compression algorithm, the millisecond-level single-byte compression time makes the computational energy consumption close to or even more than the communication energy consumption of sending the raw data. Therefore, performing a more advanced compression algorithm requires a lot of time, which may worsen the energy saving effect of compression algorithm.

As shown in Table 1, compared with the milliwatt-level transmission power of terrestrial sensor nodes [8], the power consumption of UWSNs sensor nodes is very high [1], although UWSNs nodes can adjust their transmission power like terrestrial sensor nodes. For short-range communication (<1000 m), the transmission power ensures that the data can be received as low as 5 W [1], which further reduces the communication energy consumption of nodes, whereas the computing power of nodes is up to 0.8 W. In this case, the length of the compression execution time further reduces the energy saving effect. 

Based on the above analysis, we conclude that the existing compression algorithm may not save energy in underwater applications, and only by continuously optimizing the compression algorithm can the probability of this problem be reduced. Energy optimization can be achieved through two aspects of data compression: reducing computational complexity [2] or reducing transmission power to lower the computational energy consumption occurring in local nodes. We need to consider the communication environment of the nodes to reduce the communication energy consumption of the link. Therefore, from the perspective of the whole network, applying the same algorithm to different nodes (which located at different positions), may result in different energy savings. Most of the existing energy optimization mechanisms adopt the unified compression algorithm for the whole network. It is only for specific applications and lacks adaptive ability.

For the accuracy requirements of various compression algorithms for different data applications and the different energy saving effects of network settings, such as network communication environment, and node location, etc. We propose a data upload decision-making mechanism to solve the potential compression energy saving problems. When the sensor nodes (such as the source nodes) need to send monitoring data to the sink nodes, a compression decision-making mechanism will introduced into the system under the premise of ensuring the application requirements. Nodes can choose the optimal compression strategy from the pool of compression algorithms through the decision-making mechanism to ensure the nodes transmit data packets with near-optimal energy loss and improve energy optimization.

The rest of this paper is organized as follows: In Section 2, we will briefly introduce the related research on energy optimization. In Section 3 we will show our novel data uploading strategy. Then, we proposed an evaluation of the performance of our strategy in Section 4. Finally, we present our conclusions in Section 5.

## 2. Related Work

### 2.1. Data Reduction Strategies in UWSNs

In the context of continuous monitoring, most of the data change slowly, resulting in a large amount of data redundancy in spatial and temporal. As a result, in order to limit energy consumption, reducing the amount of data without affecting data quality has become one of the most popular strategies for using data aggregation, data compression, and combinations in UWSNs [9]. Compared with terrestrial WSN, the deployment of sensor nodes in UWSNs is sparser and the typical method of reducing UWSNs data is to use sparse sampling. Compressed sensing (CS) has been thoroughly studied [9], which allows sparse analog signals to be represented by fewer samples than the Nyquist sampling theorem [10] and is essential for reducing the samples in information acquisition and sparse signal recovery [11]. CS has been adopted as the representative data reduction technique in UWSNs. Lin et al. [12] proposed an energy-efficient compression framework for UWSNs; in this framework, compressed sensing theory is applied to reduce the number of sampling nodes. Wang et al. [13] applied a cluster-based method to exploit spatial and temporal correlations for data aggregation, in which the data collected by the sensors were transmitted to the data fusion center through a hierarchy of cluster heads that used wavelet decomposition to build the wavelet coefficients of the data. Jing et al. [14] used a dynamic programming approach to decide the energy allocation ratio. Gonglaing et al. [15] proposed interleave division multiple-access based compressed sensing for information acquisition with sensor networks to ensure the data transmission reliability of information map reconstruction. Some optimization strategies were used to improve the estimation accuracy of the CS framework [16]. The optimization of the dictionary requires training samples constructed by the raw signal, but the requirement limits the recovery of the compressed signal, because the recorded raw signal recorded is unknown at the receiving terminal.

However, to date, the CS methods are mainly applied to data aggregation and processing data compression at the sink node; yet, in many cases, most of the CS methods demand accurate time synchronization and frequent network communication, resulting in huge energy overhead. This often requires powerful sensor node communication and computation capabilities, which is inefficient and impractical [12] for underwater sensor nodes, as these conditions are hard to be satisfy in common UWSNs. CS cannot be directly used for compressing raw acoustic communication data since the data are non-sparse in the temporal domain [16]. Although some works have been published about the application of CS in underwater sensor networks, few studies have focused on the problem of energy-saving transmission after compression. For the strictly computation/communication/energy limitation, the energy consumption occurring in all sensor nodes should be considered, rather than just the sink node.

Theoretically, most data reduction strategies that can be processed in the terrestrial WSNs could also be applied to UWSNs if the computation complexity satisfies the hardware conditions of UWSNs sensor node. Next, we introduce the research on the data reduction strategies in WSNs, especially the database management techniques. Finally, we introduce three data compression algorithms, which are widely applied in WSNs, including those based on temporal correlation, spatial correlation, or spatial–temporal correlation. 

### 2.2. Data Reduction Strategies in WSNs

Given the great limited sensor resources and large amount of sensed data, data management and transmission becomes more difficult, it is mandatory to introduce ways to reduce the volume of data to be transmitted. As largely detailed in [17], data reduction strategies in WSNs can generally divided into three classes: local processing algorithms [18], distributed source coding [19] and in-network data storage and query processing [20]. In-network Data Storage and Query Processing, with data being processed and stored on local node, only the end result of data analysis needs to be sent to the sink, sensor nodes in this scenario essentially form a distributed database [20,21]. In order to manage large amount of sensed data in an energy-efficient way, a database-oriented approach [20] of WSNs has proven to be useful. Bonnet et al. [22] have considered the WSNs as a relational database, in which sensor nodes represent a virtual table, having the capacity to generate continuous data streams, and which can be queried using SQL-Like queries. [23] introduced a game theoretic reward-based mechanism to balance the work load among network nodes, in which the database-oriented approach was applied to gain credit scores for nodes’ relaying service, however, since energy is highly wasted due to cooperation for data forwarding, this is inefficient and impractical for underwater sensor nodes. Haifeng et al. [24] proposed a distributed similarity search approach for retrieving the similar high dimensional sensed data in WSNs, in which a distributed LSH-based model and a distributed approximate similarity search algorithm that can be all classed inside the category of database-oriented approach were applied to compute the similarity score in the cluster head of WSNs. Note that the study design also includes how to use the large image datasets to simulate the large-scale WSNs scenario, this is inefficient and impractical for underwater scenario in which the multimedia data are hard to be gathered and processed.

Since UWSNs are application-specific and the choice of the appropriate compression methods depends on processing capability of sensor nodes and the network communication condition, the design of a distributed database-oriented in-network processing [20] mechanism adaptable to various UWSNs conditions and data similarity is necessary.

### 2.3. Temporal Domain Compression Algorithms

The temporal domain compression algorithms are typical compression algorithms based on the temporal correlation of the data. The temporal correlation indicates that the difference is small in the data that are collected by a single node over a long period. Typical temporal domain compression algorithms include the piecewise approximation algorithm [25], the predictive coding algorithm [26], and wavelet transformation [27]. The basic principle of piecewise approximation is to sequentially read the average values of the data. If the difference between the average value of the current data and that of the next sampling data exceeds a given threshold, the average value and duration of the segment are output. The prediction coding algorithm uses a certain mathematical model to obtain the predicted value of the next sampling point. If the error between the predicted value and the true value is within the predefined range, the predicted value can be substituted for the true value. Exemplary prediction coding algorithms include the autoregressive prediction algorithm and the moving average prediction algorithm. A compression technique based on curve simulation (CODST) algorithm [28] applies the regression model and curve fitting technology to realize spatial-temporal data compression. A piecewise linear approximation algorithm was proposed [29] that uses a straight line to approximate the data points that have appeared but have not been compressed under a given error bound until a new data point breaks through the bound. Similarly, starting from this new data point, new lines are used to approximate subsequent arrivals. The enabling approximate querying (EAQ) algorithm was proposed [30] that first converts the original time series into a special time series description multi-version array (MVA). With this MVA prefix, the approximate version of the original time series with certain errors can be recovered. With increasing prefix, the error decreases gradually. The time series approximation with variable error is realized. Time correlation algorithms based on time series include the discrete Fourier transform [31] and discrete wavelet transform [32].

### 2.4. Spatial Compression Algorithm

The spatial compression algorithms are based on the spatial correlation of the data. The spatial correlation reflects the similarity of the nodes with similar geographical locations at the same time. Compared with the temporal domain compression algorithm, the spatial domain compression algorithm is more complex. The cooperation between nodes should also be considered, which means that more data communication between nodes is needed. Typical spatial compression algorithms include distributed source coding DSC [33] and the Huffman algorithm [34]. Kolo et al. [33] considered data compression and routing together to eliminate the redundancy of data space as much as possible.

### 2.5. Spatial-Temporal Domain Compression Algorithm

This kind of algorithm is a combination of the temporal domain compression algorithm and the spatial domain compression algorithm. They are designed for situations in which both time and spatial correlations exist in the network data. Typical algorithms include the wavelet algorithm [35,36] and the compressed sensing algorithm [37]. The spatial–temporal wavelet transform algorithm removes the redundancy of the raw data using wavelet transform on the rows and columns of the abstract raw data matrix and achieves better compression performance with lower operational costs. An adaptive optimal zero-elimination compression algorithm was proposed that uses the spatial–temporal correlation of data to remove redundancy [38]. Based on compressed sensing theory, a data collection method with a long life cycle was designed to achieve data compression [39]. The data matrix was obtained by matching the functional relationship between the data in the dynamic time warping (DTW) path and the asynchronous data, and then compressing the data using the correlation between the data [35]. The adaptive multi-mode data compression algorithm based on wavelet transform [36] does not consider the effects of energy consumption and storage capacity of nodes, although multi-dimensional correlation is considered.

From the above analysis, most of the traditional compression algorithms are hard to directly apply to UWSNs because the existing compression algorithms used in sensor networks tend to focus on the compression performance and transmission energy consumption of the algorithms, but neglect the computational energy consumption and network performance. This poses a barrier given the limited computation and communication capacity of UWSNs nodes. Most of the existing energy optimization mechanisms adopt the unified compression algorithm for the whole network for specific applications and lack adaptive ability.

Compression algorithms vary widely, and it manages the different data types. We wanted to choose an algorithm with low complexity and good compression performance. However, the complexity and compression performance of the algorithm is compromised in design. Most of the algorithms with better compression performance are highly complex. Lower-degree algorithms usually only produce general compression performance. At present, UWSNs data acquisition mostly involves text data. Based on the redundancy characteristics of UWSNs data acquisition and the limited computing, storage, and communication capabilities of sensor nodes. Using the prediction coding, the piecewise approximation algorithm, and the wavelet transform algorithm can mine the redundancy of data time dependence, and satisfied the computation complexity. Therefore, these algorithms can be considered as an alternative compression algorithms for UWSNs.

## 3. Data Uploading Strategy

In this section, we introduce the intelligent network data uploading decision-making mechanism for application to UWSNs gateways. Based on the similarity between the data to be uploaded and the data uploaded in the previous round, the mechanism first makes a decision whether to upload the data or not. If the decision-making results need to be uploaded, the total energy that is needed by the sensor nodes to upload the data after compression needs to be further analyzed, and then the optimal upload execution strategy is selected. This strategy performs the compression operation and then directly uploads the compressed data or raw data. The goal of the proposed data upload decision-making mechanism is to ensure that the data that are uploaded in different UWSNs settings can maximize their energy savings, thereby improving the efficiency of the UWSNs energy consumption and prolonging the network’s lifetime. Data upload decisions include not uploading data, running a compression algorithm and then uploading compressed data, or uploading raw data without compression.

### 3.1. Relevant Concepts

In this section, we define the relevant concepts before a detailed description of our algorithm. 

#### 3.1.1. Compression Ratio (CR)

The CR is defined as the compressed data size over the raw data size:

(1) $$CR=\frac{D_{C}}{D_{0}}$$

where *D_c_* and *D_o_* are the size of compressed data and the raw data, respectively. The smaller the compression ratio, the better the compression performance.

#### 3.1.2. Data Similarity

Similarity [40,41] is used to describe and compare the similarity of data objects, and the value must be between 0 and 1. The higher the similarity of the objects, the larger the value. To represent the overlapping relationship between two *N*-dimensional datasets, the concept of data similarity is introduced to describe the degree of association between the two datasets. We calculated and compared the similarity of the current data to be uploaded and the previous data. The lower the value, the larger the discrepancy. The compression accuracy, which is set by all selected compression algorithms, is related to the compressed data accuracy. Then, a higher compression accuracy can be set to obtain accurate current data that have larger discrepancies from the uploaded data. To compute the energy consumption that results from the compression computation, we set the data similarity to be the compression accuracy of the selected compression algorithm, and finally we obtained the CR through the calculation.

The data similarity in our algorithm is only related to the data value, including the intersecting dimensionality between data sets and the corresponding overlapping proportion in each dimension. Its characteristics are as follows:

(1) When the overlapping ratio in each dimension remains unchanged, the greater the intersecting dimensionality, the higher the similarity.
(2) When the intersecting dimensionality between data sets remains unchanged, the larger the overlapping ratio in each dimension, the higher the similarity.

With the discussion above, we define the similarity of two data sets in dimension i as:

(2) $$dos_{i}=\sqrt{\left|\sin\left(\frac{\pi }{2}*\left|\frac{x_{i1}-x_{i2}}{y_{i1}-y_{i2}}\right|\right)\right|}$$

where $y_{1}$ and $y_{i2}$ denote the thresholds of the unions of the *i*-th attribute in the two data sets, which indicate the size of the union proportion; $x_{i1}$ and $x_{i2}$ denote the thresholds of the intersection of the *i*th attribute in the two data sets, which indicate the size of the intersecting proportion.

To compare the similarity between the two datasets and eliminate the influence of dimensions, $dos_{i}$ is normalized as:

(3) $$dos_{i}=\frac{dos_{i}-\underset{1\leq j\leq n}{\min\{dos_{j}\}}}{\underset{1\leq j\leq n}{\max\{dos_{j}\}}-\underset{1\leq j\leq n}{\min\{dos_{j}\}}}$$

where *n* denotes the total number of dimensions. After $dos_{i}$ is derived, we define the similarity of the two data sets as the average of the similarities in all dimensions:

(4) $$dos=\sum_{i=1}^{n}dos_{i}/n.$$

### 3.2. Joint Power Control and Rate Adaptation Algorithm

In this section, we propose an algorithm that combines the power control and the rate adjustment. The mechanism is based on the channel gain information and allows as much parallel transmission as possible by using a game theory-based Nash equilibrium solution [42]. To effectively protect marine animals, the mechanism introduces a dual-mode access control strategy based on the location results [43]. When sharing the spectrum with the marine biological system, excessive transmission power is avoided. Through the above power control algorithm, by implementing the power control scheme, we allow for as many concurrent transmissions as possible. However, the Signal to Interference plus Noise Ratio (SINR) at the receiver side changes significantly after the transmission power is adjusted. To manage the signal variations, the transmission rate should be tuned accordingly. In other words, modems with a higher SINR can choose a more aggressive mode with a higher data rate; otherwise, modems can choose a more conservative mode with a lower data rate to achieve lower bit error rate (BER) performance. Finally, to ensure a certain bit error rate requirement, we increase the transmission rate to reduce the network’s energy consumption as much as possible. This mechanism provides the optimal transmission power and the transmission rate for the data upload decision-making mechanism in this paper. The details are provided below. 

#### 3.2.1. Power Control Algorithm

Considering the presence of marine animals or other acoustic systems around the current network, the sending node avoids impacting on these systems when calculating the transmit power. Therefore, the algorithm adopts a dual-mode power control mechanism that includes a sharing mode and an exclusive mode.

##### **Sharing** **Mode**

If the network has detected some marine animals, the sensor nodes in the network switch to the sharing mode to control their transmission power. In this mode, when a node request sending a packet, its transmission power should not be higher than the threshold that can affect the behavior of marine animals. In this method, we adopt the behavioral interruption threshold (160 dB re µPa) as the threshold. In networks, each node is considered selfish but rational, which means that they want to maximize their own interests. Therefore, the transmission power allocation problem can be treated as a game that can be solved by using the game theory. To fulfill the goals, a utility function that contains both a utility term and a pricing term is defined:

(5) $$u_{i}(p_{i},P_{-1})=\log(1+\frac{h_{i}\cdot p_{i}}{\sum_{j=1}^{N-1}h_{j}\cdot p_{j}+\sigma^{2}})-\alpha_{i}\cdot p_{i}$$

where *p_i_* is the transmission power on the link *i*, $h_{i}$ is the channel gain in channel *i*, $p_{-i}$ is the transmission power on other links except link i, N is the total number of the UWSNs links, $\alpha_{i}$ (bit/watt) is the bit price for one unit of transmission power, and $\sigma^{2}$ denotes the noise power (variance). We assumed that every node shares the same bandwidth *B*. Based on this assumption, *B* was omitted when we constructed the utility function. The first part of the utility function denoting the relationship between the level of sending power and the corresponding link’s capacity is based on Shannon theory [44]. The second part of the utility function reflects the price of consuming a certain amount of power.

Finally, the power allocation problem and the substitution are formulated, as shown in Equations (6) and (7), respectively:

(6) $$maxu_{i}(p_{i},P_{-1})=\log(1+\frac{h_{i}\cdot p_{i}}{\sum_{j=1}^{N-1}h_{j}\cdot p_{j}+\sigma^{2}})-\alpha_{i}\cdot p_{i}$$

(7) $$\begin{array}{l} s.t.\\ p_{i}\in [0,P_{\max}](1\leq i\leq N)\\ \frac{h_{i}\cdot p_{i}}{\sum_{j=1}^{N-1}h_{j}\cdot p_{j}+\sigma^{2}}\geq SINR_{th}(1\leq i\leq N)\\ \sum_{i=1}^{N}h_{mi}\cdot p_{i}\leq T \end{array}$$

where $p_{\max}$ max is the maximum sending power that is limited by the hardware’s design, $SINR_{th}$ is the decoding threshold of the acoustic modem, T is the behavioral interruption threshold, and $h_{mi}$ is the channel gain between the sender and marine mammals. The third constraint in Equation (7) indicates that the receiving power from all senders should not be larger than the behavioral interruption threshold (160 dB re μPa) [45].

From this discussion, the sensor’s power allocation problem is an optimization problem; therefore, the optimal power allocation problem can be solved by using the Nash equilibrium (NE) [42].

##### **Exclusive** **Mode**

If the network does not detect any marine animals, the sensor nodes will transmit their packets in exclusive mode. In this mode, the aim of environmentally friendly power control (EFPC) is to maximize the network throughput while reducing the energy consumption. Thus, we formulated the optimization problem (Equation (8)) and that with two limitations (Equation (9)).

(8) $$maxu_{i}(p_{i},P_{-1})=\log\left(1+\frac{h_{i}\cdot p_{i}}{\sum_{j=1}^{N-1}h_{j}\cdot p_{j}+\sigma^{2}}\right)-\alpha_{i}\cdot p_{i}$$

(9) $$\begin{array}{l} s.t.\\ p_{i}\in [0,P_{\max}](1\leq i\leq N)\\ \frac{h_{i}\cdot p_{i}}{\sum_{j=1}^{N-1}h_{j}\cdot p_{j}+\sigma^{2}}\geq SINR_{th}(1\leq i\leq N) \end{array}$$

The only difference compared with the share mode is that the third constraint, which is used for limiting the impacts on marine animals, is deleted. Through a similar deduction, the optimal power allocation problem can also be solved by using the NE.

#### 3.2.2. Rate Adaptation Algorithm

Since an Orthogonal Frequency Division Multiplex (OFDM) modem is capable of working in five modes, the working modes [46] of OFDM modems are essentially decided by the current SINR, which reflects the current channel condition. In other words, with a higher SINR [1], modems can choose a more aggressive mode with a higher data rate. Otherwise, modems will choose a more conservative mode with a lower data rate. Inspired by this conclusion, combined with the bit error rate (BER) requirement at the receiver side, we propose a rate adaptation method for an underwater OFDM modem:

(10) $$R_{t}=\{\begin{array}{l} 0,\;SINR<\beta_{0}\\ M_{k},\;\beta_{k-1}\leq SINR\leq \beta_{k},\left(1\leq k\leq 4\right)\\ M_{5},\;\beta_{5}\leq SINR \end{array}$$

where $R_{t}$ is the adopted transmission mode, $M_{k}$ is one of the five modes of the OFDM modems, and $\beta_{k}\cdot (\beta_{k-1})$ is the upper (lower) threshold for setting the modem mode to $M_{k}$.

### 3.3. Data Upload Decision-Making Mechanism

**Figure 3:**
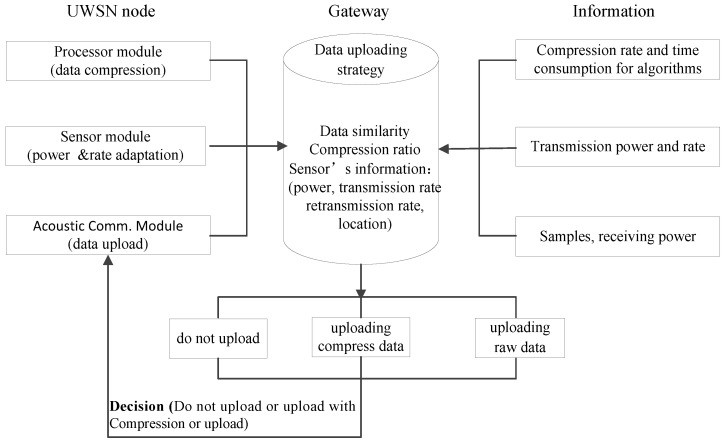
Architectural diagram of the data upload decision-making mechanism.

When the UWSNs node needs to upload the collected data to the gateway, the data upload decision-making mechanism is introduced into the gateway system, and the gateway executes the data upload decision-making mechanism. The architecture is shown in Figure 3.

As seen in Figure 3, the information that is required by the gateway to perform the data uploading decisions including: the similarity between the current data to be uploaded and the previous data; the compression ratio obtained by the alternative compression algorithm; and the transmission power, reception power, calculation power, data transmission rate, data retransmission rate, and source node location of the related nodes. This information is mainly provided by the three modules of sensor nodes. The node transmission power and its data transmission rate are obtained by performing the joint power control and rate adjustment algorithm that was described in Section 3.2. The algorithm can select the appropriate transmission power and data transmission rate according to the existence of animals and the channel conditions. The data retransmission rate is obtained by calculating the average retransmission rate of the data that were uploaded in the previous *i-th* rounds. The data similarity is obtained by calculating the similarity between the data to be uploaded in this round and the data from the previous round using the gateway processing unit, which was set as the precision requirement of each alternative compression algorithm, and the corresponding compression ratio and compression time are calculated. The receiving power and computing power are determined by the node’s hardware. Based on the above information, the gateway makes the UWSNs data upload decisions with the goal of maximizing the energy savings of the integrated network. If the decision-making results no need to be uploaded, the decision-making process is completed; if the decision-making results need to be uploaded, the total energy consumption of the uploaded compressed data, including the node compression calculation’s energy consumption and the total energy consumption of directly uploading the raw data, need to be compared. The sensor node executes an alternative compression algorithm according to the accuracy requirement or uploads the raw data directly without compression. Finally, the decision results are fed back to the source nodes.

**Figure 4:**
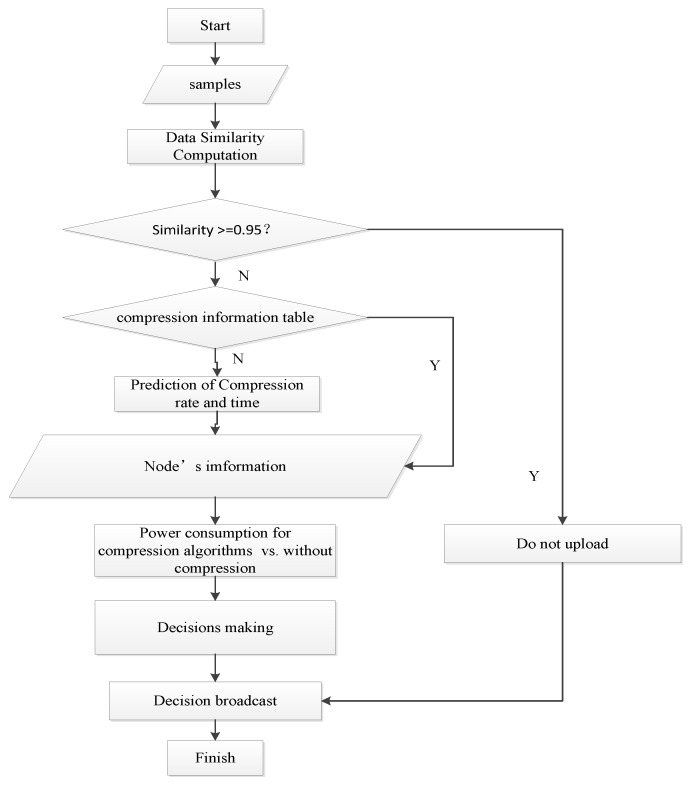
Flow chart of the data upload decision-making mechanism.

To acquire historical information of the data similarity and compression algorithms for the data uploading decision-making mechanism, a two-dimension table was established to store the compression information of each underwater node, which is listed as {node number, data similarity, compression algorithms, average CR, average compression time, retransmission}. The proposed mechanism needs to compute the energy consumption of the data compression and transmission for each node. Figure 4 shows the communication process and describes the mechanism in steps.

Step 1: Carry out similarity calculation. Calculate the similarity of the 500 current data to be uploaded and the 500 previous data, which were sampled chronologically and expressed as $ds=\{\left(t_{1},d_{1}),(t_{2},d_{2}),\ldots (t_{500},d_{500}\right)\},\;\mathrm{where}\;(t_{i},d_{i})$ denotes the sampled data at one time. Next, calculate the similarity of the current sampled data and the previous sampled data. If the value is greater than 0.95, decide that the current data do not need to be uploaded due to the high data similarity between the current data and the previous data. Then, proceed to the step 8 and end this upload decision-making; otherwise, the mechanism continues to step 2.

Step 2: In this step, the source node number and data similarity query in the compression information table are completed. If the corresponding record cannot be found, then continue to step 3; otherwise, proceed to step 4.

Step 3: The prediction of the CR and compression time. In this step, the CR and the compression time for one byte of data (*T_nc_*) that can meet the precision requirement of each alternative compression algorithm are calculate. Then, this calculation result is written into the compression information table.

Step 4: Perform the joint power control and rate adjustment algorithm that are described in Section 3.2 to determine the proper sending power and data transmission rate.

Step 5: The gateway obtains the relevant information of the nodes that are needed for the data upload decision-making mechanism (“Predictive information", Figure 3).

Step 6: The total energy consumption of the uploaded compressed data is calculated and compared, including the node compression calculation’s energy consumption and the total energy consumption to directly uploading the raw data. Then, select the optimal upload execution strategy with the lowest total energy consumption. Finally, broadcast this selected strategy to the source node.

The total energy consumption of the compression and uploaded compressed data includes two parts: the compression calculation’s energy consumption and the transmission’s energy consumption including the energy consumption of sending and the energy consumption of receiving. The total energy consumption of directly uploading the raw data only includes the transmission’s energy consumption. The compression calculation’s energy consumption is formulated using Equation (11), the energy consumption of sending is calculated using Equation (12), and the energy consumption of receiving is calculated using Equation (13):

(11) $$E_{nc}=P_{nc}*L*T_{nc}(dos)$$

(12) $$E_{ns}=\sum_{i=1}^{h}P_{ns-i}*L*CR\left(dos\right)*T_{n-i}*\left(1+{rs}_{i}\right)$$

(13) $$E_{nr}=\sum_{i=1}^{h-1}P_{ns-i}*L*CR\left(dos\right)*T_{n-i}*\left(1+{rs}_{i}\right)$$

As such, the total energy consumption of the uploaded compressed data after compression can be calculated using:

(14) $$E_{cp}=E_{nc}+E_{ns}+E_{nr}$$

Then, the total energy consumption of directly uploading the raw data without compression includes the energy consumption of sending (*E*_*ns*−*un*_) and the energy consumption of receiving $\left(E_{nr-un}\right)$, which can be calculated using Equations (15) and (16), respectively:

(15) $$E_{ns-un}=\sum_{i=1}^{h}P_{ns-i}*L*T_{n-i}*\left(1+{rs}_{i}\right)$$

(16) $$E_{nr-un}=\sum_{i=1}^{h-1}P_{nr-i}*L*T_{n-i}*\left(1+{rs}_{i}\right)$$

After $E_{ns-un}$ and $E_{nr-un}$ are derived, the total energy consumption of directly uploading the raw data can be calculated using:

(17) $$E_{uncp}=E_{ns-un}+E_{nr-un}$$

When h=1, $E_{cp}$ and $E_{uncp}$ can be calculated using Equations (18) and (19), respectively:

(18) $$\begin{array}{l} E_{cp}=P_{nc}*L*T_{nc}\left(dos\right)+P_{ns}*L*\\ CR\left(dos\right)*T_{n}*\left(1+rs\right) \end{array}$$

(19) $$E_{uncp}=P_{ns}*L*T_{n}*\left(1+rs\right)$$

where h, $P_{nc}$, and L denote the total number of hops from the source node to the gateway, the compression calculation power, and the raw data size, respectively; *dos* represents the data similarity requirements; $T_{nc}$ is the average time cost for one byte of data compression under *dos*; $P_{ns-i}$ and $P_{nr-i}$ denote the sending power of the sending node and the receiving power of the receiving node at the *i*th hop of the transmission path, respectively; CR is the compression ratio that is obtained by the alternative compression algorithm under $dos\cdot T_{n-i}$, which is the average time cost for transmitting one byte of data, and determined by the transmission rate at the *i*-th hop; and ${rs}_{i}$ denotes the data retransmission rate of the sending node at the *i*-th hop of the transmission path. We did not consider the energy consumption of receiving of the gateway. Therefore, the transmission’s energy consumption does not include the energy consumption of receiving when h=1.

Sep 7: Select the minimum values of $E_{cp}$ and $E_{uncp}$ and the corresponding strategy as the data upload decision. This decision could provide better energy saving results for the UWSNs.

Step 8: End the upload decision-making process.

## 4. Simulation and Evaluation

**Figure 5:**
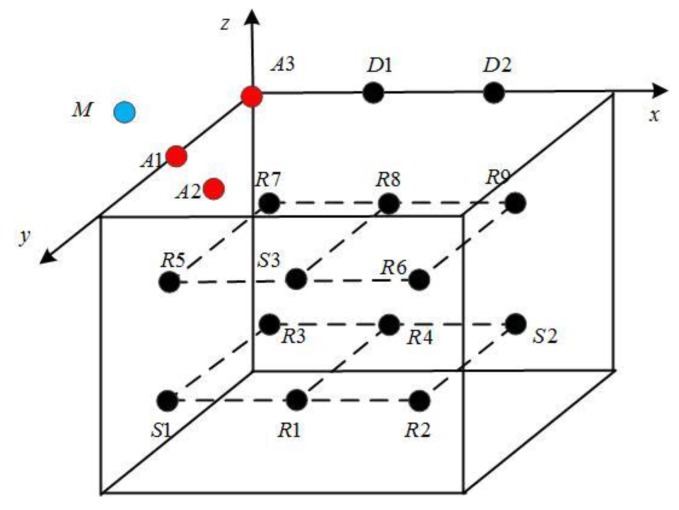
Network topology.

In this section, we verify the energy optimization function of the proposed data upload decision-making mechanism for a UWSNs. The network topology is shown in Figure 5, where A1–A3 are the three anchor nodes for locating sensor nodes and animals. Among them, A1 and A3 are deployed on the water surface, A2 is set to 250 m below the water surface. D1and D2 are the gateway nodes (sink nodes), deployed on the water surface, that receive the data from the sensor nodes and are responsible for receiving and collecting data. Suppose nodes S1–S3 are the source nodes of the data transmission and R1–R9 are the relay nodes, among which S1, S2, R1, R2, R3, and R4 are set to 1500 m below the water surface; and S3, R5, R6, R7, R8, and R9 are set to 500 m below the water surface. They are deployed in the range of 2000 × 3000 × 2000 m, and the range is divided into 12 1000 × 1000 × 1000 m grids. Each sensor node is placed in the center of a 1000 × 1000 × 1000 m three-dimensional cube space; the communication range is set to 1000 m. We use *M* to represent an animal that is detected traveling at a speed of *V* (1,0,0). We selected 10 groups of data in chronological order from the measured data of the temperature, salinity, and dissolved oxygen in one week near an island located in Sanya, Hainan province, China. Each group contained 50 sample data, the packet length was 100 B, and all data were normalized to [0, 1].

Three temporal domain compression algorithms, greedy disconnected piecewise linear approximation (GDPLA) [47], the first-order autoregression [48], and the 5/3 wavelets [49], were selected as the compression algorithm options for the data upload decision-making mechanism. The data upload decision-making mechanism that is proposed in this section is not limited to specific data types and the alternative compression algorithms mentioned here. The following simulation sets the data upload decision result to be uploaded (data similarity is less than 0.95). The compression ratios of different alternative compression algorithms under different data similarity requirements are shown in Table 2. 

**Figure 6:**
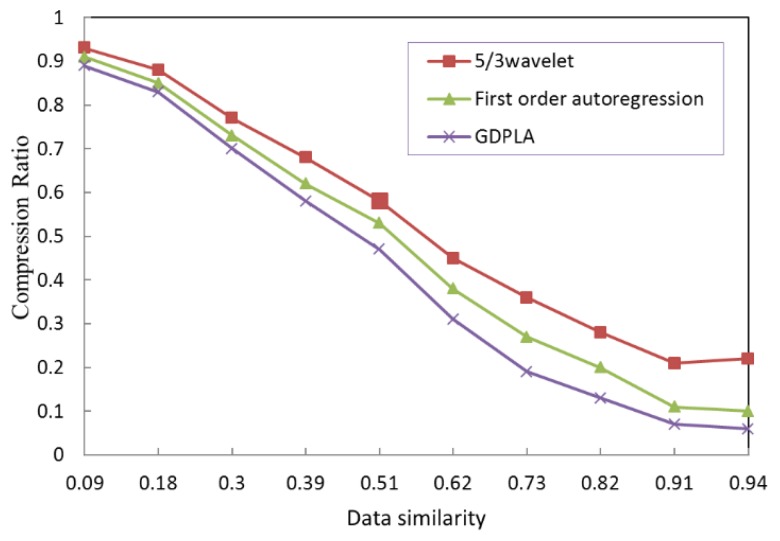
Similarities and compression ratios of the compression algorithms.

To intuitively evaluate the compression performance of each alternative compression algorithm, Figure 6 depicts the CR with varying data similarities. This figure shows that the accuracy requirement that is represented by the data can similarity lowers the CR. When monitoring the data of a single node, there is such a relationship: the higher the data similarity, the lower the CR, and the better the compression performance. As shown in Figure 6, the GDPLA and first-order autoregressive prediction that the higher algorithmic complexities can produce better compression performance.

**Figure 7:**
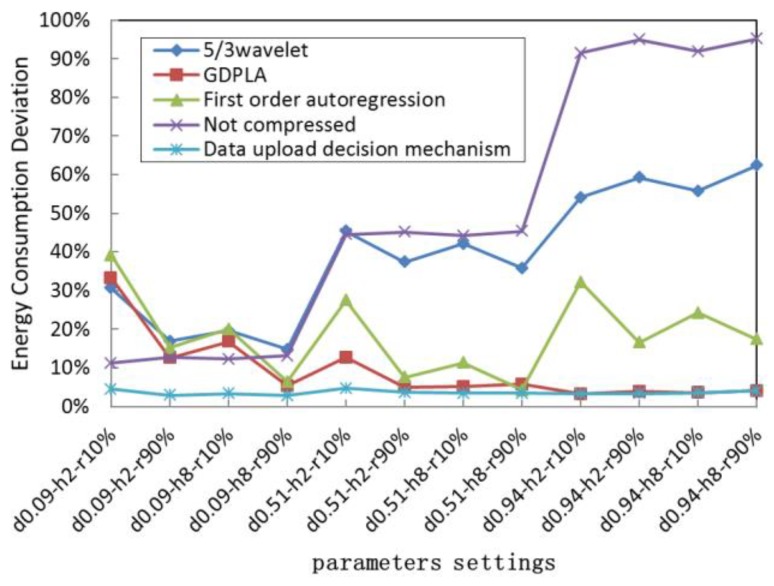
Deviation between the energy consumption of the upload strategies and the optimal energy consumption.

To evaluate the energy saving effect of different data upload decision-making strategies, we calculated the deviation between the total energy consumption of different upload strategies and the optimal energy consumption [50] of different UWSNs nodes in different network settings. The simulation results are listed at Table 3, and Figure 7 shows the energy saving deviation.

As shown in Table 3, the network settings of our simulation include three data similarities (compression accuracy requirement), two numbers of hops, and two data retransmission ratios, which represent different compression accuracy requirements, distances nearer or farther from the gateway, and better or worse underwater acoustic channel quality, respectively. For example, the parameter setting in the first row shows the data similarity is 0.09, the number of hops away from the gateway is 2, and the data retransmission is 90% respectively.

In Figure 7, the parameter setting on x-axis d0.09-h2-r90% shows that the data similarity (d) is 0.09, the number of hops away from the gateway (h) is 2, and the data retransmission ratio (r) is 90%.

We evaluated the energy consumption performance of the method that applies our proposed data upload decision-making strategy. For comparison, we also evaluated the energy consumption performance of other alternative compression algorithms. As shown in Figure 7, all nodes can upload data with optimal energy consumption performance in different network settings. We have further analyzed the impact of the data similarity and transmission distance on data uploading energy consumption.

Table 3 shows that due to the time-varying characteristics of the underwater channel, uploading compressed data does not necessarily save more energy than uploading data without compression. Determining whether to run an alternative compression algorithm and then uploading the compressed data is also related to the link quality of the transmission path and the data similarity. In detail, when the data similarity is low, the energy consumption of compression calculation is large, resulting in the total energy consumption of the uploaded compressed data being higher than the total energy consumption of the uploaded uncompressed raw data. Therefore, the source node close to the gateway will choose the compression strategy with no compression or low complexity. With the increase in the distance, the energy consumption of communication increases exponentially. At this time, even with the energy consumption of the compression calculation, the total energy consumption of data upload after compression is still lower than that of the raw data upload without compression. Therefore, the nodes choose the algorithm with a better compression effect to compress and upload data. When the data similarity is higher, which means significantly lower energy consumption and better compression performance, the source nodes will choose to perform compression and upload the compressed data after the operation of decision-making.

**Figure 8:**
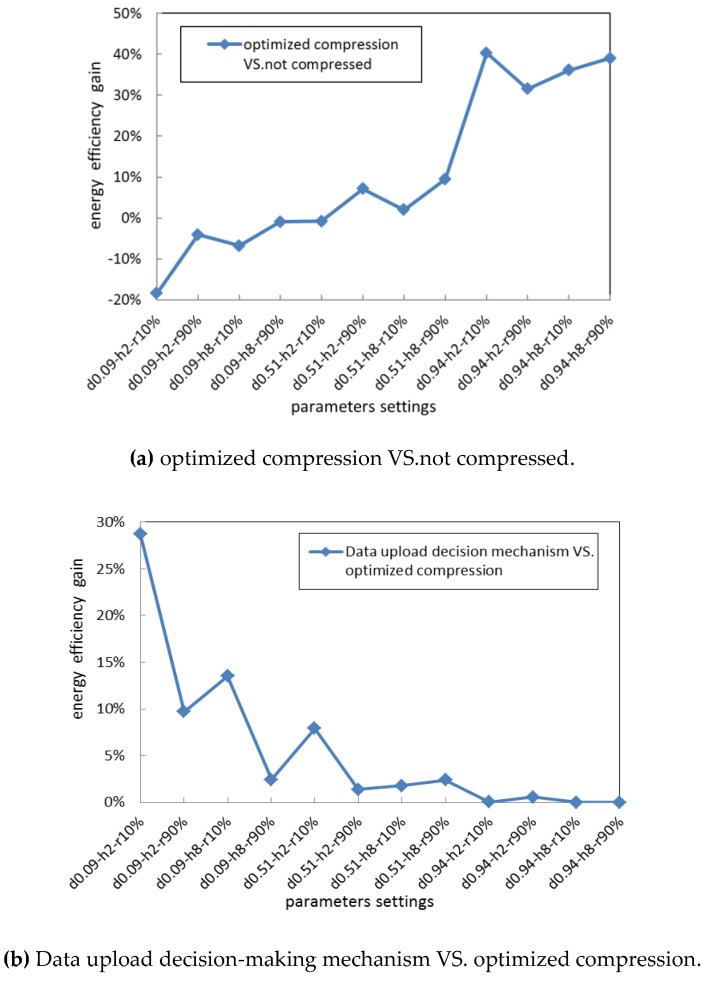
Energy efficiency gain.

We compared the network energy efficiency gain when using the unified optimized compression algorithm with better compression performance, against when the data are not compressed or our proposed data upload decision-making mechanism is used. The simulation results are shown in Figure 8.

Figure 8a shows that when the data similarity is lower or the source nodes are closer to the gateway, the optimized compression can obtain negative energy efficiency gain, mainly because the higher compression computation energy consumption could result in the total energy consumption required for uploading the compressed data exceeding the total energy consumption required for directly uploading the data without performing any compression. Instead, as shown in Figure 8b, our proposed data upload mechanism, which selects an adaptive upload strategy according to the quality of network communication and the requirement of compressed precision, can obtain higher energy efficiency gain against when the unified and optimized compression algorithm is used. For all the network settings above, we know that our proposed data upload decision-making strategy can upload the data with relatively lower energy consumption.

## 5. Conclusions

A large similarity problem exists in underwater sensor network data acquisition, with considerable amounts of redundant data in the network. Due to the quality of the network communication, the data compression algorithm that is used in the UWSNs may not save energy. Considering the limited energy available in underwater networks, to avoid wasting energy due to uploading compressing duplicate and redundant data, a data upload decision-making mechanism for underwater networks with the goal of optimizing energy consumption was proposed in view of the high similarity redundancy of the collected data. By analyzing the data similarity and the uploading energy consumption, the mechanism provides the underwater node with the best decision-making strategy for data upload. Compared with the single compression algorithm, the data upload decision-making mechanism proposed in this paper can achieve better energy savings. 

## Figures and Tables

**Table 1:** Power parameters of underwater nodes.

Data Rate (kbps)	1–10
Processing power (W)	<0.8
Transmission power (W)	<35

**Table 2:** Similarities and compression ratios of the compression algorithms.

**Data similarity**	0.09	0.18	0.30	0.39	0.51	0.62	0.73	0.82	0.91	0.94
**5/3 wavelet**	0.93	0.88	0.77	0.68	0.58	0.45	0.36	0.28	0.21	0.22
**First-order autoregression**	0.91	0.85	0.73	0.62	0.53	0.38	0.27	0.20	0.11	0.10
**GDPLA**	0.89	0.83	0.70	0.58	0.47	0.31	0.19	0.13	0.07	0.06

**Table 3:** Deviation between the energy consumption of the upload strategies and the optimal energy consumption.

Compression Accuracy	No. Hops	Retransmission Ratio	5/3 Wavelet	GDPLA	First-Order Autoregression	Not Compressed	Data Upload Decision-Making Mechanism
0.09	2	10%	30.67%	33.25%	39.18%	11.26%	4.51%
0.09	2	90%	16.89%	12.57%	15.24%	12.66%	2.89%
0.09	8	10%	19.62%	16.81%	20.03%	12.29%	3.31%
0.09	8	90%	14.82%	5.27%	6.41%	13.11%	2.85%
0.51	2	10%	45.36%	12.64%	27.48%	44.52%	4.71%
0.51	2	90%	37.34%	4.97%	7.56%	45.18%	3.59%
0.51	8	10%	42.07%	5.16%	11.34%	44.19%	3.38%
0.51	8	90%	35.84%	5.79%	4.26%	45.34%	3.41%
0.94	2	10%	54.11%	3.23%	32.16%	91.46%	3.21%
0.94	2	90%	59.24%	3.86%	16.59%	94.92%	3.32%
0.94	8	10%	55.78%	3.51%	24.27%	91.89%	3.51%
0.94	8	90%	62.32%	4.02%	17.39%	95.16%	4.01%

## References

[1] Wan L., Zhou H., Xu X., Huang Y., Zhou S., Shi Z., Cui J.H. (2015). Field tests of adaptive modulation and coding for underwater acoustic OFDM. IEEE J. Ocean. Eng..

[2] Lin J.W., Liao S.W., Leu F.Y. (2019). Sensor Data Compression Using Bounded Error Piecewise Linear Approximation with Resolution Reduction. Energies.

[3] Zhu L. (2018). Research on WSN Data Compression Based on Predictive Class Aglorithm. Master’s Thesis.

[4] Shuang Z., Zhihong Q., Xiaohui L. (2016). Data Compression Algorithm Based on Sequence Correlation for WSN. J. Electron. Inf. Technol..

[5] Chen S., Lu J. (2013). An Adaptive Multiple-Modality Sensor Network Data Compression Algorithm Based on Lifting Wavelet and Polynomial Fitting. Chin. J. Sens. Actuators.

[6] Zhang R., Du S., Chen L., Kan J., Xu G. (2015). Data Compression Method with Piece-Wise Linear Regression in WSN. Chin. J. Sens. Actuators.

[7] Atmel Corporation AVR Studio 4. http://www.atmel.com/dyn/Products/tools_card.asp?tool_id=2725.

[8] Li C., Wang J., Li M. (2017). An Efficient Cross-Layer Optimization Algorithm for Data Transmission in Wireless Sensor Networks. Int. J. Wirel. Inf. Netw..

[9] Adcock B., Hansen A.C. (2016). Generalized sampling and infinite-dimensional compressed sensing. F Found. Comput. Math..

[10] Jianhua Q., Xueying Z. (2017). Compressed sensing based data gathering in wireless sensor networks: A survey. J. Comput. Appl..

[11] Liu B., Zhang Z. (2016). Quantized Compressive Sensing for Low-Power Data Compression and Wireless Telemonitoring. IEEE Sens. J..

[12] Lin H., Wei W., Zhao P., Ma X., Zhang R., Liu W., Deng T., Peng K. (2016). Energy-efficient compressed data aggregation in underwater acoustic sensor networks. Wirel. Netw..

[13] Wang D., Xu R., Hu X., Su W. (2016). Energy-Efficient Distributed Compressed Sensing Data Aggregation for Cluster-Based Underwater Acoustic Sensor Networks. Int. J. Distrib. Sens. Netw..

[14] Jing L., He C., Huang J., Ding Z. (2017). Energy Management and Power Allocation for Underwater Acoustic Sensor Network. IEEE Sens. J..

[15] Liu G., Kang W. (2014). IDMA-Based Compressed Sensing for Ocean Monitoring Information Acquisition with Sensor Networks. Math. Probl. Eng..

[16] Wu F.Y., Yang K., Duan R., Tian T. (2018). Compressive Sampling and Reconstruction of Acoustic Signal in Underwater Wireless Sensor Networks. IEEE Sens. J..

[17] Li S., Kim J.G., Han D.H., Lee K.S. (2019). A Survey of Energy-Efficient Communication Protocols with QoS Guarantees in Wireless Multimedia Sensor Networks. Sensors.

[18] Taherpour A., Chobin M., Rahmani M. (2019). Collaborative data aggregation using multiple antennas sensors and fusion centre with energy harvesting capability in WSN. IET Commun..

[19] Rane S., Cohen R.A., Vetro A., Sugimoto K. (2015). Method for Improving Compression Efficiency of Distributed Source Coding Using Intra-Band Information. U.S. Patent.

[20] Diallo O., Rodrigues J.J.P.C., Sene M., Lloret J. (2015). Distributed Database Management Techniques for Wireless Sensor Networks. IEEE Trans. Parallel Distrib. Syst..

[21] Diallo O., Rodrigues J.J., Sene M., Lloret J. (2014). Simulation framework for real-time database on WSNs. J. Netw. Comput. Appl..

[22] Bonnet P., Gehrke J., Seshadri P. (2001). Towards Sensor Database Systems. Proceedings of the International Conference on Mobile Data Management.

[23] Umar M.M., Khan S., Ahmad R., Singh D. (2018). Game Theoretic Reward Based Adaptive Data Communication in Wireless Sensor Networks. IEEE Access.

[24] Hu H., He J., Wu J., Wang K., Zhuang W. (2017). Distributed high-dimensional similarity search approach for large-scale wireless sensor networks. Int. J. Distrib. Sens. Netw..

[25] Lazaridis I., Mehrotra S. (2003). Capturing sensor-generated time series with quality guarantees. Proceedings of the 19th International Conference on Data Engineering (Cat. No.03CH37405).

[26] Yan H., Shi Z., Cui J.H. DBR: Depth-Based Routing for Underwater Sensor Network. Proceedings of the 2018 International Conference on Research in Networking.

[27] Pham M.L., Ramstad T.A., Balasingham I. (2009). Ultra wideband biomedical wireless sensor networks using wavelet lifting for image transmission. Proceedings of the 2009 International Conference on Information Processing in Sensor Networks.

[28] Wang L., Ma C. (2010). A one-dimensional linear regression model based spatial and temporal data compression algorithm for wireless senor networks. J. Electron. Inf. Technol..

[29] Elmeleegy H., Elmagarmid A.K., Cecchet E., Aref W.G., Zwaenepoel W. (2009). Online piece-wise linear approximation of numerical streams with precision guarantees. VLDB Endow..

[30] Yu L., Li J., Gao H., Fang X. (2009). Enabling ε-Approximate Querying in Sensor Network. Proc. VLDB Endow..

[31] Fazel F., Fazel M., Stojanovic M. (2011). Random access compressed sensing for energy-efficient underwater sensor networks. IEEE J. Sel. Areas Commun..

[32] Siripanadorn S., Hattagam W., Teaumroong N. (2010). Anomaly detection in wireless sensor networks using self-organizing map and wavelets. Int. J. Commun..

[33] Pattem S., Krishnamachari B., Govindan R. (2008). The impact of spatial correlation on routing with compression in wireless sensor networks. ACM Trans. Sens. Netw..

[34] Kolo J.G., Ang L.M., Shanmugam S.A., Lim D.W.G., Seng K.P. (2013). A Simple Data Compression Algorithm for Wireless Sensor Networks. Soft Computing Models in Industrial and Environmental Applications.

[35] Zhou S.W., Lan L.I. (2014). DTW-based multi-wavelet data compression algorithm for wireless sensor networks. J. Commun..

[36] Zhu T.J., Lin Y.P., Zhou S.W., Xu X.L. (2009). Adaptive multiple-modalities data compression algorithm using wavelet for wireless sensor networks. J. Commun..

[37] Luo C., Wu F., Sun J., Chen C.W. (2009). Compressive data gathering for large-scale wireless sensor networks. Proceedings of the 15th Annual International Conference on Mobile Computing and Networking.

[38] Jiang P., Wu J.F., Wu B., Dong L.X., Wang D. (2013). Data compression method for wireless sensor networks based on adaptive optimal zero suppression. J. Commun..

[39] Chen Z., Yang G., Chen L., Zhou Q. (2014). Data gathering for long network lifetime in WSNs based on compressed sensing. J. Electron. Inf. Technol..

[40] Ji J., Pang W., Zhou C. (2012). A fuzzy k-prototype clustering algorithm for mixed numeric and categorical data. Knowl. Based Syst..

[41] Wu X., Chen G. (2018). Avoiding energy holes in wireless sensor networks with non-uniform node distribution. IEEE Trans. Parallel Distrib. Syst..

[42] Pottier A., Socheleau F.X., Laot C. (2016). Power-efficient spectrum sharing for noncooperative underwater acoustic communication systems. Proceedings of the OCEANS 2016 MTS/IEEE Monterey.

[43] Yao G., Jin Z., Su Y. An environment-friendly spectrum decision strategy for underwater wireless sensor networks. Proceedings of the 2015 IEEE International Conference on Communications.

[44] Caspers E.P., Yeung S.H., Sarkar T.K. (2013). Analysis of Information and Power Transfer in Wireless Communications. IEEE Antennas Propag. Mag..

[45] Janik V.M. (2000). Source levels and the estimated active space of bottlenose dolphin (Tursiops truncatus) whistles in the Moray Firth, Scotland. J. Comp. Physiol. A.

[46] Yan H., Zhou S., Shi Z.J., Li B. A DSP implementation of OFDM acoustic modem. Proceedings of the Second Workshop on Underwater Networks.

[47] Li J., Li G., Gao H. (2015). Novel E-Approximation to Data Streams in Sensor Networks. IEEE Trans. Parallel Distrib. Syst..

[48] Zhang Y., Chen H., Xu W., Yang T.C., Huang J. (2017). Spatiotemporal Tracking of Ocean Current Field with Distributed Acoustic Sensor Network. IEEE J. Ocean. Eng..

[49] Ding L., Chen W. The Analysis and Research of Lifting Scheme based on Wavelet Transform. Proceedings of the International Conference on Cyber Security Intelligence and Analytics.

[50] Cao B., Zhao J., Lv Z., Liu X., Kang X., Yang S. (2018). Deployment optimization for 3D industrial wireless sensor networks based on particle swarm optimizers with distributed parallelism. J. Netw. Comput. Appl..

